# Preparation of a platinum electrocatalyst by coaxial pulse arc plasma deposition

**DOI:** 10.1088/1468-6996/16/2/024804

**Published:** 2015-03-27

**Authors:** Yoshiaki Agawa, Hiroyuki Tanaka, Shigemitsu Torisu, Satoshi Endo, Akihiro Tsujimoto, Narishi Gonohe, Victor Malgras, Ali Aldalbahi, Saad M Alshehri, Yuichiro Kamachi, Cuiling Li, Yusuke Yamauchi

**Affiliations:** 1 Arc Plasma Deposition System Business Promotion Division, ULVAC-RIKO, Inc., 4388 Ikonobe-cho, Tsuzuki, Yokohama 224-0053, Japan; 2 International Center for Materials Nanoarchitectonics (MANA), National Institute for Materials Science (NIMS), 1-1 Namiki, Tsukuba, Ibaraki 305-0044 Japan; 3 Department of Chemistry, College of Science, King Saud University, Riyadh 11451, Saudi Arabia

**Keywords:** Pt nanoparticles, electrocatalysts, methanol oxidation

## Abstract

We have developed a new method of preparing Pt electrocatalysts through a dry process. By coaxial pulse arc plasma deposition (CAPD), highly ionized metal plasma can be generated from a target rod without any discharged gases, and Pt nanoparticles can be deposited on a carbon support. The small-sized Pt nanoparticles are distributed over the entire carbon surface. From transmission electron microscopy (TEM), the average size of the deposited Pt nanoparticles is estimated to be 2.5 nm, and their size distribution is narrow. Our electrocatalyst shows considerably improved catalytic activity and stability toward methanol oxidation reaction (MOR) compared with commercially available Pt catalysts such as Pt black and Pt/carbon (PtC). Inspired by its very high efficiency toward MOR, we also measured the catalytic performance for oxygen reduction reaction (ORR). Our PtC catalyst shows a better performance with half-wave potential of 0.87 V, which is higher than those of commercially available Pt catalysts. The higher performance is also supported by a right-shifted onset potential. Our preparation is simple and could be applied to other metallic nanocrystals as a novel platform in catalysis, fuel cells and biosensors.

## 1. Introduction

Recently, direct methanol fuel cells (DMFCs) have been attracting much attention as efficient power sources because of their high energy density, low operation temperature, low pollutant emission and ease of handling liquid fuel [1–5]. Platinum (Pt) and its alloys are the best catalysts for DMFCs. Due to platinum’s high price and limited natural resources, the Ministry of Economy, Trade and Industry (Japan) points to the following measures in the road map: (i) reduction of platinum, (ii) core–shell catalyst of platinum and (iii) development of a substitute for the platinum catalyst. In the current research, the rational synthesis of nanostructured Pt is of great importance for the development of next-generation catalysts [6–10]. The catalytic activity of nanostructured Pt is highly dependent on the surface area, surface atomic structures and crystal size and shape [11–14]. By controlling the size and shape of nanostructured Pt, large surface area with abundant catalytic active sites can thereby favor enhanced catalytic performance and lead to the desired utilization efficiency of Pt [15–18]. The synthesis of Pt nanoparticles is currently attracting considerable interest toward the realization of high-performance Pt catalysts. Various general and effective routes have been developed to synthesize nanostructured Pt materials [11, 19–22]. Conductive supports, such as activated carbon and nickel foam, are usually used to support Pt nanoparticles with enhanced activity and improved stability [23–25]. Wet process has been mostly utilized for Pt/carbon (PtC) catalysts [23, 24].

CAPD is a dry process for depositing metal nanoparticles on substrates. The CAPD method enables one to easily generate nanometer-sized particles on any kind of substrate [26–28]. In this work, we successfully deposited small-sized Pt nanoparticles on carbon supports to prepare novel PtC electrocatalysts. It is quite interesting that small-sized Pt nanoparticles are distributed over the entire carbon surface. From TEM, the average size of the deposited Pt nanoparticles is estimated to be 2.5 nm, and their particle size distribution is narrow. Here, we evaluated the catalytic activity of our PtC catalyst toward MOR and ORR. Our PtC performance is higher than commercially available PtC catalysts.

## 2. Experimental section

### 2.1. Preparation of the Pt electrocatalyst by CAPD

**Figure 1.**
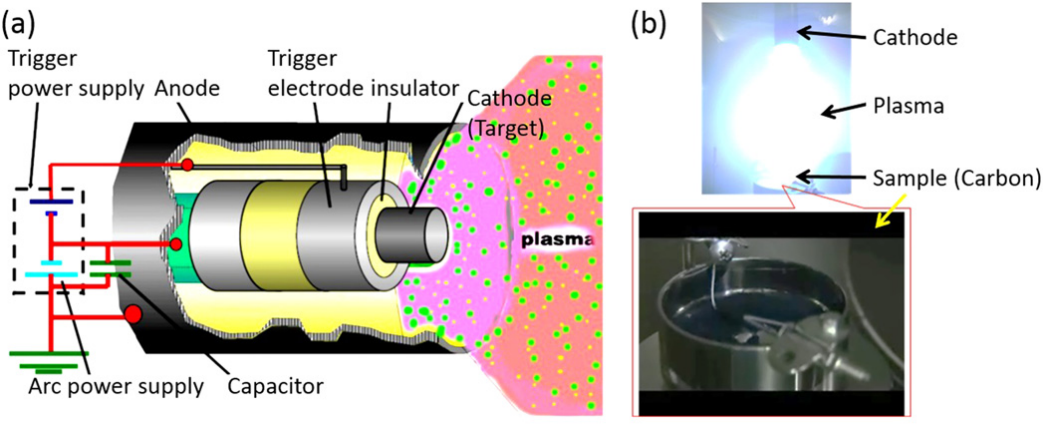
Experimental set-up for preparation of the PtC catalyst prepared by CAPD. (a) Illustration of the generation of an arc plasma. (b) Photographs of an arc plasma and a sample holder.

Our experimental setup is shown in figure 1(a). Carbon powder (Ketjenblack carbon: 20 cc) is stored in a vessel (*φ*80 mm × 50 mm) made of stainless steel (figure 1(b)). This vessel is set at the center of a vacuum chamber (1.3 × 10^**−**3^ Pa). The cathode is set at 80–100 mm from the top of the cup. The operational parameters of CAPD are the condenser capacity (1080 *μ*F), discharge voltage (150 V) and discharge count (ca. 28 958 shots). Finally, our Pt catalyst containing 5 wt% Pt and 95 wt% carbon support is obtained. Hereafter, the as-made sample is abbreviated as ‘PtC-CAPD’.

### 2.2. Electrochemical analysis

#### 2.2.1. MOR measurements

The cyclic voltammograms (CVs) and chronoamperometric curves were analyzed with a CHI 842B electrochemical analyzer (CH Instrument, USA). A conventional three-electrode cell was used, including a Ag/AgCl electrode as a reference electrode, a platinum wire as a counter electrode and a glassy carbon electrode (GCE, 3 mm in diameter) modified with catalyst as a working electrode. Prior to the surface coating, the GCE was polished carefully with a 1.00 and 0.05 *μ*m alumina powder and rinsed with deionized water, followed by drying with nitrogen gas. After that, the samples were coated on the surface of the GCE. Here, our PtC-CAPD electrocatalyst was compared to three commercially available catalysts: Pt black (PtB, Alfa-Aesar), Pt (5%) on carbon (PtC-5%, Alfa-Aesar) and Pt (20%) on carbon (PtC-20%, Alfa-Aesar). The Pt loading amount of each sample is: 2 *μ*g (for our Pt-CAPD catalyst), 5 *μ*g (for PtB), 2 *μ*g (for PtC-5%) and 4 *μ*g (for PtC-20%). Then, the Nafion solution (5.0 *μ*L, 0.5 wt%) was coated on the surface and dried completely at room temperature. Before MOR, a cyclic voltammetric measurement ranging from −0.2 to 1.5 (versus Ag/AgCl) at a scan rate of 500 mV · s^−1^ was carried out for 100 cycles to clean the electrode. MOR investigation was carried out in a 0.5 M H_2_SO_4_ solution containing 0.5 M methanol. After obtaining the CV curves, chronoamperometric measurement (*i*–*t*) was measured for 2000 s by applying a constant of 0.5 V. All potentials were referenced to a Ag/AgCl (saturated KCl) electrode.

### 2.3. ORR measurements

The polarization curves for the ORR were carried out with a CHI 842B electrochemical analyzer (CH Instrument, USA). A conventional three-electrode cell was used, including a Ag/AgCl electrode as a reference electrode, a platinum wire as a counter electrode and a rotation disk electrode (RDE, 4 mm in diameter) modified by catalyst as a working electrode. The working electrode was prepared as follows: 5 mg of catalyst was dispersed in a mixture containing 950 *μ*L of ethanol/H_2_O (3:1 volume ratio) and 50 *μ*L Nafion (5 wt%) under ultrasonication for 30 min in order to obtain a uniform suspension. Then, 5 *μ*L of the suspension was dropped on the surface of the RDE and dried under room temperature. The loading amount of each sample on the electrode was 20 *μ*g. The electrochemical measurements were performed in basic media (0.1 M KOH), saturated with O_2_, in a potential window ranging from −1.0 and 0.0 V (versus Ag/AgCl) with a scan rate of 10 mV · s^−1^.

## 3. Result and discussions

**Figure 2.**
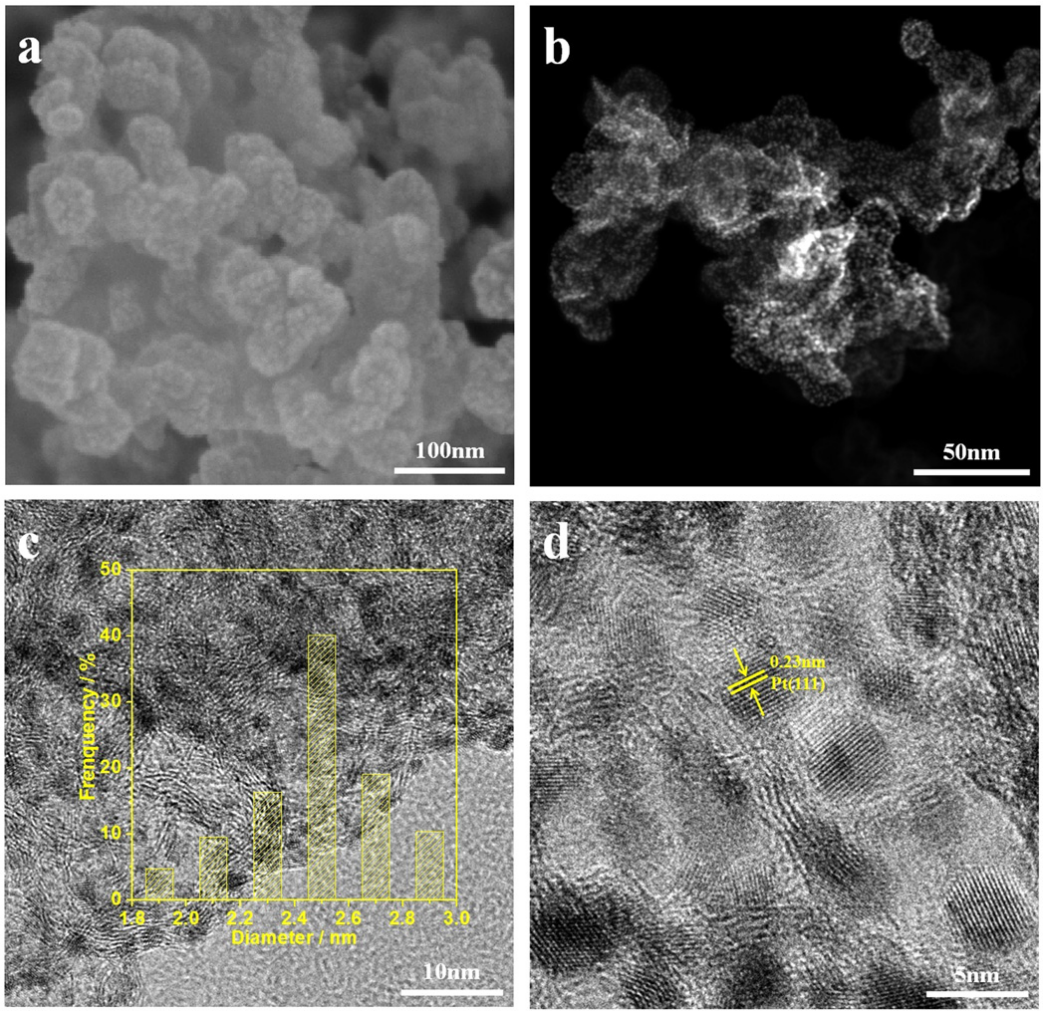
(a) SEM image, (b) HAADF-STEM image and (c) and (d) bright-field TEM images of Pt nanoparticles (5 wt%) deposited on carbon support. The corresponding histogram of the particle size distribution is also shown as the inset in panel (c).

A scanning electron microscope (SEM) image of the PtC catalyst prepared by CAPD is shown in figure 2(a). The surface topography indicates that small-sized Pt nanoparticles are distributed over the entire carbon surface. In figure 2(b), the high angle annular dark field scanning transmission electron microscopy (HAADF-STEM) image shows the location of the Pt nanoparticles, which are mostly deposited on the carbon surface. To further characterize the deposit, the samples were observed by high-resolution transmission electron microscopy (HRTEM). As seen from the bright-field TEM image (figure 2(c)), the average size of the deposited Pt nanoparticles is estimated to be 2.5 nm, and their particle size distribution is narrow. Each deposited particle is well crystalized. The observed *d* spacing of 0.23 nm between two adjacent fringes can be assigned to the (111) diffraction planes of the face-centered cubic *fcc* Pt crystal structure (figure 2(d)) [29, 30]. The electronic state of Pt was characterized by x-ray photoelectron spectroscopy (XPS). The position of the doublet peaks at binding energies of 74.4 eV and 71.0 eV can be assigned to the Pt^0^ 4*f*_5/2_ and Pt^0^ 4*f*_7/2_ components, respectively, which indicates the formation of metallic Pt.

**Figure 3.**
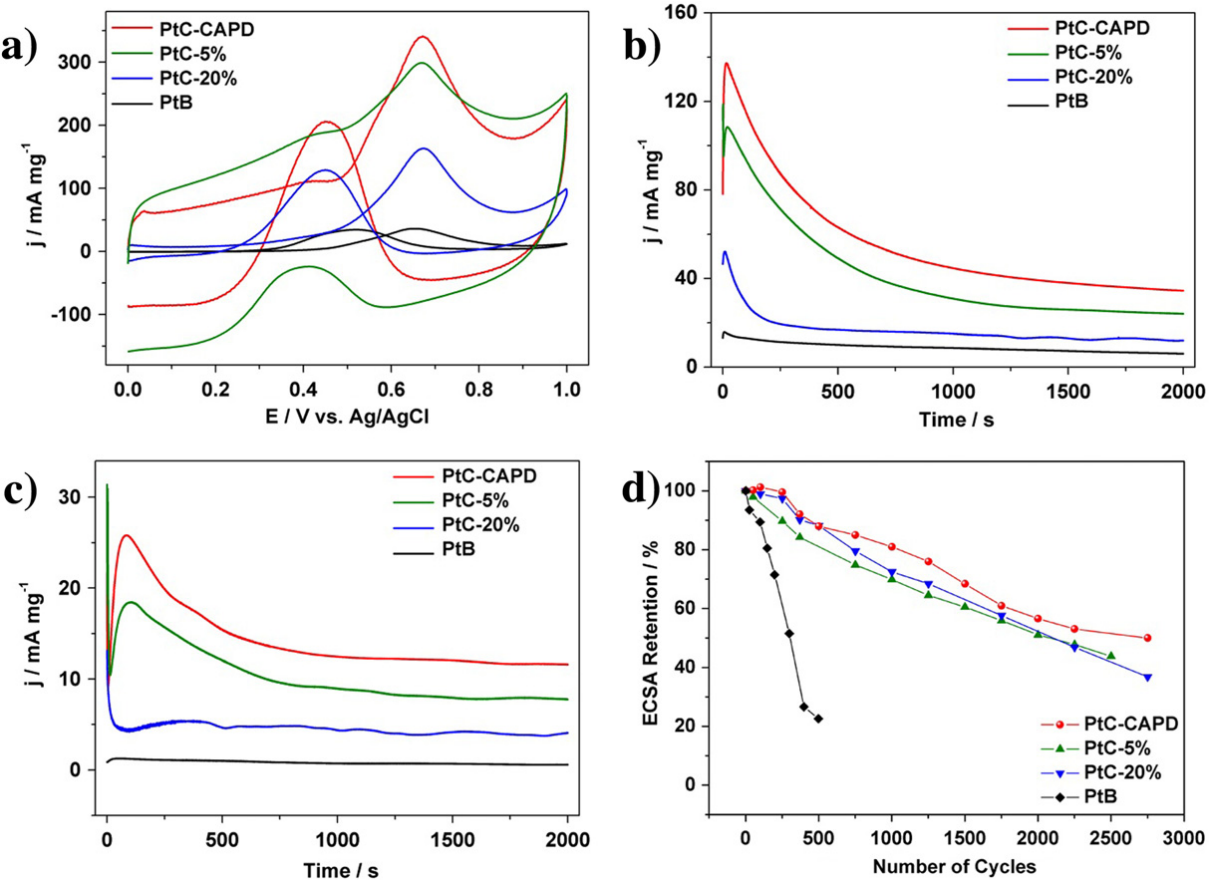
(a) Cyclic voltammetric and (b), (c) amperometric curves for the MOR catalyzed PtC-CAPD, PtC-5%, PtC-20% and PtB in a 0.5 M H_2_SO_4_ solution containing 0.5 M methanol. (d) Pt_ECSA retention of each sample during the cycling treatments. The current densities (*Y*-axis) are normalized by the mass of Pt (mg). A scanning speed of 50 mV s^−1^ is used for the (a) cyclic voltammetric measurements, while constant potentials of 0.5 V and 0.4 V are used in the (b), (c) amperometric measurements.

The electrocatalytic activity of MOR was studied. The reaction for the entire MOR can be written as follows: CH_3_OH + H_2_O → CO_2_ + 6 H^+^ + 6e^−^. The first reaction step is written as CH_3_OH → CO_ad_ + 4 H^+^ + 4e^−^. At the early stage, the methanol molecules are adsorbed to the Pt surface, and CO molecules are generated, which is described as CO_ad_. At the second step, water molecules are adsorbed to the Pt surface. The water molecules adjacent to CO_ad_ are reactive to generate CO_2_ molecules (CO_ad_ + OH^−^ → CO_2_ + H^+^ + 2e^−^) [31–34]. For comparison, several commercially available Pt catalysts (PtB, PtC) with different Pt loading amounts (PtC-5% and PtC-20%) were also evaluated. Figure 3(a) shows the CV curves of the MOR catalyzed by different Pt catalysts. All the CV curves exhibit two noticeable anodic peaks, which are typical features of a MOR on a pure Pt surface [35–37]. In the case of our Pt catalyst and PtC-5%, an electric double-layer region is also observed within which the carbon support has a significant contribution. The area of the electric double-layer varies according to the surface area of the carbon supports. The current density of different Pt catalysts of the MOR is summarized in table 1. Our Pt catalyst shows higher current density than other commercially available catalysts.

**Table 1.** Comparison of the peak currents of each sample MOR at different stages.

*j* (mA mg^−1^)	PtC-CAPD	PtC-5%	PtC-20%	PtB
Initial	341.0	298.8	163.2	36.1
After *i*–*t* at 0.5 V	335.2	274.4	140.0	24.0
After *i*–*t* at 0.4 V	303.3	262.8	128.6	15.5

**Figure 4.**
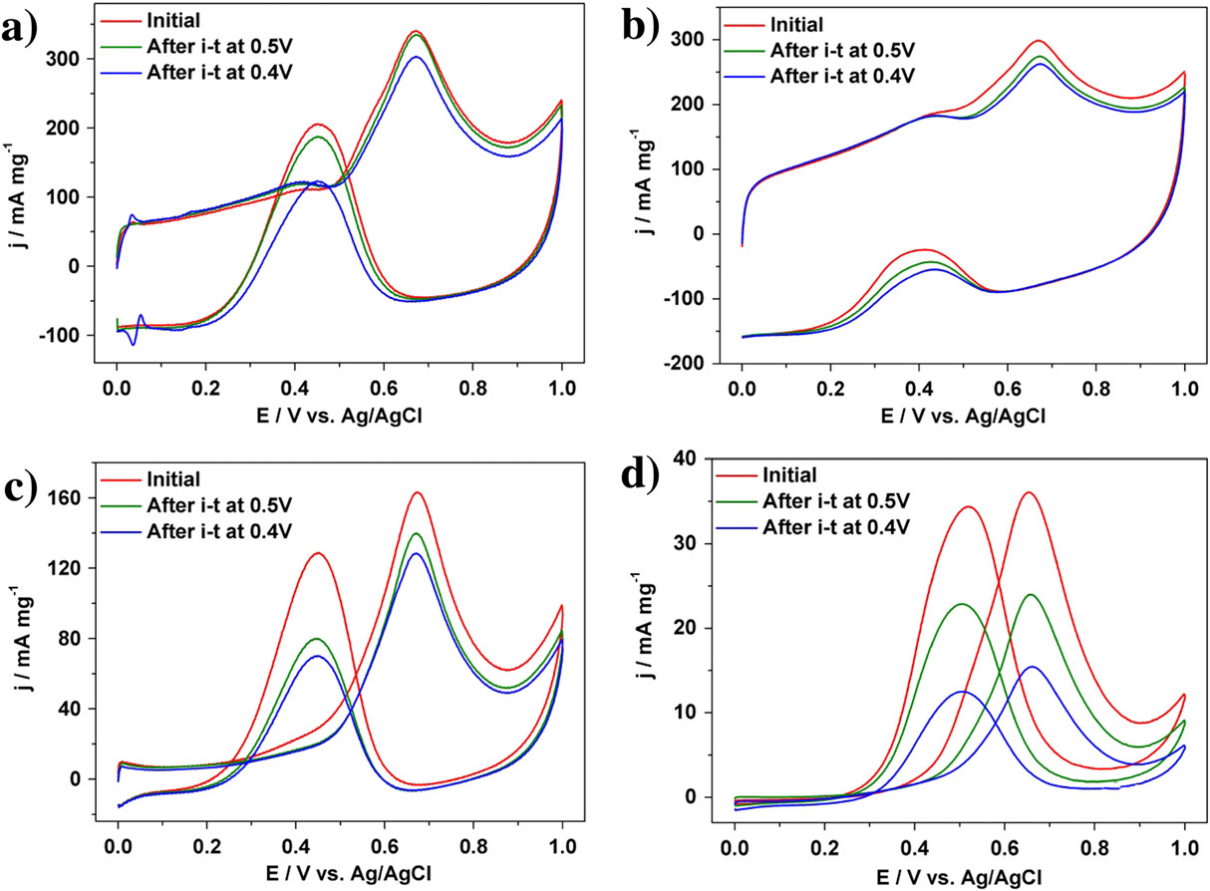
Cyclic voltammograms for MOR catalyzed (a) PtC-CAPD, (b) PtC-5%, (c) PtC-20% and (d) PtB catalysts, respectively, in a 0.5 M H_2_SO_4_ solution containing 0.5 M methanol at different stages.

In addition to the catalytic activity, the durability of Pt catalysts is also a very important factor for practical usage. Both the activity and the durability were investigated by using chronoamperometric curves at 0.5 V for 2000 s (figure 3(b)). The current density of our Pt catalyst than the other samples. For each sample, the initial current density gradually decreased, and finally reach a plateau with a stable current value. In addition to the highest initial activity, our Pt catalyst retains superior current density even after 2000 s (table 1). Even when the applied potential was changed to 0.4 V, the activities of different Pt catalysts are in the same order (figure 3(c)). After 2000 s, MOR performance was evaluated (figure 4, table 1). As clearly seen from table 1, when the potential is 0.4 V, MOR activity loss is only 11.1% for the PtC-CAPD catalyst, which is significantly lower compared to PtB (57.1%), PtC-5% (12.0%) and PtC-20% (21.2%). This further testifies that our Pt catalyst exhibits superior electrocatalytic performance of the MOR.

To better understand the reason for such high activity and stability, we further studied the Pt electrochemically active surface area (ECSA) loss for each sample. All the stability measurements were conducted in 0.5 M H_2_SO_4_ at a potential ranging from 0.6 to 1.0 V, which causes the surface oxidation/reduction cycles of Pt. After a certain period, the CV plot was recorded in the potential range of −0.2 V to 1.0 V to calculate the Pt ECSA. The Pt ECSA of each sample is estimated by integrating the charge during hydrogen adsorption/desorption on the electrode surface by assuming that the charge required to oxidize a monolayer of hydrogen on the Pt surface is 210 *μ*C · cm^−2^. Before the test, we evaluated the initial ECSA for each catalyst. Our Pt catalyst shows the highest ECSAs (75.4 m^2^ · g^−1^), which is higher than 12.4 m^2^ · g^−1^ (PtB), 64.9 m^2^ · g^−1^ (PtC-5%), and 66.8 m^2^ · g^−1^ (PtC-20%). The CV curves after different cycles were recorded, and the ECSA loss of each sample was plotted, as shown in figure 3(d). After 2000 cycles, the ECSAs of our Pt catalyst show less than a 50% loss compared to PtC-5% (56.2%), PtC-20% (63.2%) and PtB (77.4%). It is worth noting that the ECSA of our Pt catalyst is firstly increased then decreased compared to the initial stage, which is consistent with the mass transport limitation caused by the high surface area of the carbon support (e.g. Ketjenblack) [38, 39].

We consider that a good adhesion exists between the fine Pt nanoparticles and the carbon support, which is effective to good stability [40, 41]. The carbon support (Kenjenblack) employed in the current work possesses a rough surface which is beneficial to the Pt loading. The formation of Pt seeds and the growth of Pt nanoparticles on the carbon support proceed under an arc charge condition, as shown in figure 1. Therefore, the adhesion of Pt nanoparticles will be largely improved, unlike other methods. Both the dissolution of Pt nanoparticles through the Pt oxidation and the corrosion of the carbon support are known to be common issues that affect activity and stability [42–44]. Our CAPD method is a promising new method which addresses this matter.

**Figure 5.**
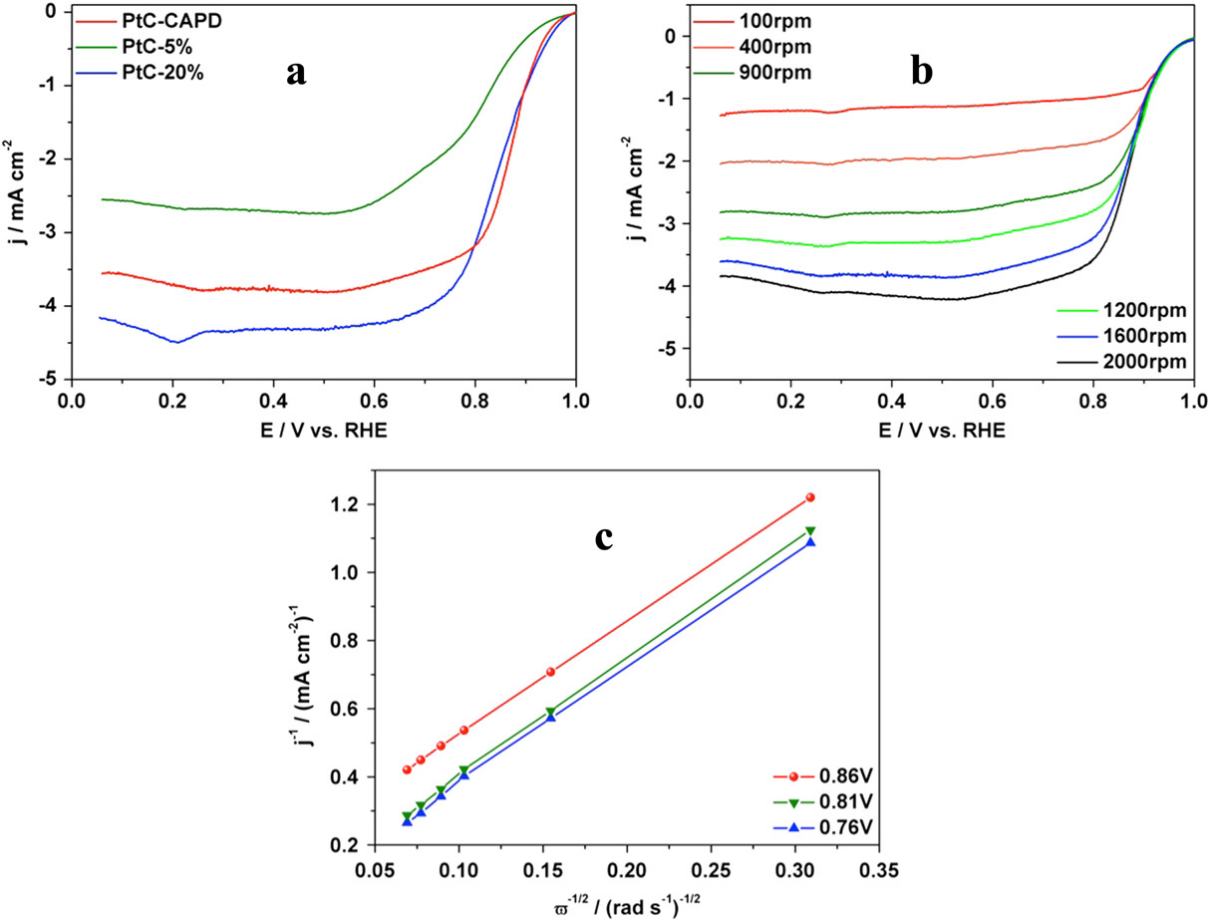
(a) ORR polarization curves of a RDE modified with PtC-CAPD, PtC-5% and PtC-20%. The plots were obtained in O_2_-saturated KOH (0.1 M) at a rotation rate of 1600 rpm with a scan rate of 10 mV s^−1^. (b) ORR polarization curves of PtC-CAPD at different rotation rates (from 100 rpm to 2000 rpm) in O_2_-saturated KOH (0.1 M) with a scan rate of 10 mV s^−1^. (c) The Koutecky–Levich (K–L) plots of PtC-CAPD at various potentials. RHE stands for reversible hydrogen electrode.

Inspired by its very high efficiency toward MOR, the catalytic performance toward ORR was analyzed in order to get more evidence for practical application in fuel cell technology. The sluggish oxygen reduction and the low efficiency of catalysts are usually the two main problems for fuel cell applications. Here, our Pt catalyst was evaluated and compared with other carbon-supported Pt catalysts (PtC-5% and PtC-20%). The typical polarization plots of each sample are displayed in figure 5(a). All samples show the oxygen reduction activity and the diffusion-controlled current plateaus. Compared to the two commercially available PtCs, our catalyst shows a better performance with a half-wave potential of 0.87 V, which is 0.09 and 0.03 V more positive than those of PtC-5% and PtC-20% catalysts, respectively. The higher performance was also supported by the higher onset potential. To determine the ORR electron-transfer pathway, the polarization curves of PtC were recorded at different rotation rates. An increase of the limiting diffusion current densities as a function of the rotation rate was obtained (figure 5(b)). The corresponding Koutecky–Levich (K–L) plots are shown in figure 5(c); the electron transfer number of 3.88 suggests that our PtC sample catalyzes the ORR mainly through a four-electron process [45, 46].

## 4. Conclusions

We have developed a new method of preparing Pt electrocatalysts through a dry process. A highly ionized metal plasma was generated and small-sized Pt nanoparticles were successfully deposited on a carbon support. Uniformly-sized Pt nanoparticles with an average size of 2.5 nm are distributed over the entire carbon surface. Our PtC shows considerably improved catalytic activity and stability toward MOR compared with commercially available Pt catalysts such as PtB, PtC-5% and PtC-20%. Our CAPD method showed a good adhesion between the fine Pt nanoparticles and the carbon support, which is necessary to realize stable catalysts. Inspired by its very high efficiency toward MOR, the catalytic performance toward ORR shows better performance with the half-wave potential of 0.87 V, which is more positive than those of commercially available Pt catalysts. The higher performance is also supported by the right-shifted onset potential. Our preparation is useful as a simple and green approach, which could be applicable to other metallic nanocrystals and carbon supports (e.g. nanoporous carbon [47–49]) as a novel platform in catalysis, fuel cells and biosensors.
